# Kardiale Magnetresonanztomographie: Trends und Entwicklungen

**DOI:** 10.1007/s00117-020-00766-3

**Published:** 2020-11-05

**Authors:** A. Mayr, G. Reiter, D. Beitzke

**Affiliations:** 1 Universitätsklinik für Radiologie, Medizinische Universität Innsbruck, Anichstraße 35, 6020 Innsbruck, Österreich; 2 Research and Development, Siemens Healthcare Diagnostics GmbH, Straßgangerstraße 315, 8054 Graz, Österreich; 3 Universitätsklinik für Radiologie und Nuklearmedizin, Medizinische Universität Wien, Währinger Gürtel 18–20, 1090 Wien, Österreich

**Keywords:** T1-Mapping, 4‑Flussmessungen, Speichererkrankungen, Myokarditis, Compressed sensing, T1 mapping, 4‑D flow measurements, Myocardial storage diseases, Myocarditis, Compressed sensing

## Abstract

**Hintergrund:**

Durch die Entwicklung robuster Techniken und deren umfassender Validierung hat sich die kardiale Magnetresonanztomographie (CMR) in ihrem knapp 25-jährigen klinischen Einsatz ein breites Indikationsspektrum erarbeitet. Die Erfassung kardialer Volumina und systolischer Ventrikelfunktion sowie die Charakterisierung fokaler Myokardnarben sind heute Teil der CMR-Standard-Bildgebung. Zuletzt haben die Einführung beschleunigter Bildakquisitionstechnologien, die neuen Bildgebungsmethoden des myokardialen T1- und T2-Mappings und der 4‑D-Flussmessungen sowie die neue Postprocessing-Technik des myokardialen Feature-Trackings an Relevanz gewonnen.

**Methode:**

Diese Überblicksarbeit basiert auf einer umfassenden Literaturrecherche in der PubMed-Datenbank zu neuen CMR-Techniken und ihrer klinischen Anwendung.

**Ergebnisse und Schlussfolgerung:**

Dieser Artikel zeigt eine Übersicht über die neuesten technischen Entwicklungen im Bereich der CMR sowie deren Anwendungsmöglichkeiten anhand der wichtigsten klinischen Fragestellungen.

## Abstract (en)

**Background:**

In its almost 25 years of clinical use, cardiac magnetic resonance imaging (CMR) has been developed for a wide range of indications due to the development of robust techniques and their comprehensive validation. CMR-based assessment of cardiac volumes and systolic ventricular function as well as the characterization of focal myocardial scars belongs today to standard cardiac imaging. More recently, the introduction of accelerated acquisition techniques, quantitative myocardial T1- and T2-mapping methods and 4‑dimensional (4D) flow measurements as well as new postprocessing techniques such as myocardial feature tracking have attracted attention.

**Methods:**

This review is based on a comprehensive literature search in the PubMed database on new CMR techniques and their clinical application.

**Results and conclusion:**

This article provides an overview of the latest technical developments in the field of CMR and their possible applications based on the most important clinical MR issues.

Aktuelle Entwicklungen in der kardialen Magnetresonanztomographie (CMR) haben zuletzt einige Limitationen konventioneller, etablierter CMR-Sequenzen überwunden. Beschleunigte Bildakquisitionstechniken wie „compressed sensing“, die Möglichkeit der absoluten Quantifizierbarkeit auch diffuser myokardialer Veränderungen mittels myokardialem Mapping oder neue Postprocessing-Anwendungen wie „strain imaging“ erweitern die Effizienz und den klinischen Nutzen der CMR in einer Vielzahl von Herzerkrankungen [1]. Mittlerweile finden sich in 54 % aller Leitlinien der europäischen kardiologischen Gesellschaft (ESC Guidelines) Empfehlungen zur Anwendung der CMR [2].

## Neueste technische Entwicklungen

### Beschleunigte Bildrekonstruktion

**Figure 1:**
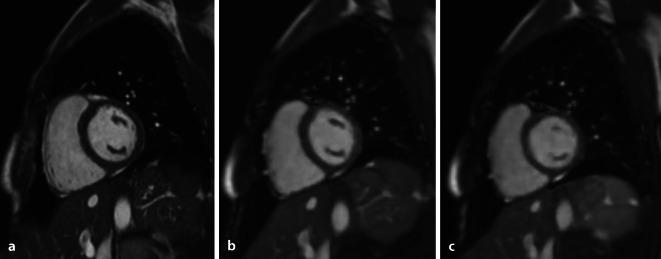

Für die in segmentierter Weise zeitintensiv erfassten CMR-Bilder wird jeweils ein Teil der erforderlichen k‑Raumlinien in unterschiedlichen Herzschlägen während einer Atemanhaltephase EKG-synchronisiert akquiriert. Diese Technik reagiert empfindlich auf Arrhythmien und Atemanhalteprobleme. Die in den letzten Jahren entwickelten Ansätze zur Verkürzung der CMR-Bildakquisition beruhen auf der Rekonstruktion eines vollen Bildes aus vorherigen, stark unterabgetasteten, jedoch mit mehreren Spulenelementen aufgenommenen Dateninformationen im k‑Raum. Die parallelen Bildgebungstechniken kommerzieller Magnetresonanztomographie(MRT)-Systeme wie Sensitivity Encoding (Sense) und Generalized Autocalibrating Partial Parallel Acquisition (Grappa) sind typischerweise auf eine 2‑ bis 3‑fache Beschleunigung beschränkt. Die innovative Compressed-sensing(CS)-Technik ermöglicht eine Bildrekonstruktion aus noch deutlich weniger Datenzeilen, indem der Informationsgehalt der CMR-Bilder komprimiert wird („sparsity“) und rauschartige Bildrekonstruktionsartefakte durch eine iterative Bildrekonstruktion eliminiert werden [3]. Die CS-Technik findet in unterschiedlichen Sequenzen Anwendung, darunter in der Perfusionsbildgebung [4], der Echtzeit-Cine-Bildgebung unter freier Atmung ([5]; Abb. 1), der Phasenkontrast-Flussmessung [6], dem 3‑D-Late-Gadolinium-Enhancement [7] und im T1-Mapping [8]. Rezente Arbeiten zeigen eine CS-Anwendung auch auf kontinuierlich erfasste radiale Techniken in freier Atmung [9]; dabei können Daten sowohl in Herz- als auch in Atemdimensionen getrennt werden, ohne dass ein Herz-Gating oder ein Anhalten des Atems erforderlich ist. Die zunehmende Verfügbarkeit verbesserter Computerhardware macht diese rechenintensiven Rekonstruktionsmethoden nun praktisch anwendbar. Nachdem zahlreiche Untersuchungen die Robustheit der CS-Techniken für die routinemäßige klinische Anwendung zeigen konnten, werden diese Methoden in den aktuellen Generationen kommerzieller MRT-Systeme zur Verfügung gestellt.

### Erweiterte Myokardcharakterisierung

Die Late-Gadolinium-Enhancement(LGE)-Technik stellt die etablierteste CMR-Methode zur Charakterisierung fokaler Myokardveränderungen dar [10]. Die dabei verwendeten T1-gewichteten Sequenzen differenzieren mit hoher diagnostischer Genauigkeit anhand des Gadolinium-Verteilungsmusters im extrazellulären Myokardkompartiment ischämische von nichtischämischen Pathologien und liefern relevante prognostische Informationen [11]. Wesentliche Nachteile dieser Technik liegen in der fehlenden Unterscheidbarkeit zwischen akuten und chronischen Veränderungen sowie in der Erfassung diffuser myokardialer Schädigungen. Als spannendste CMR-Neuerung bieten die zuletzt in die klinische Routine eingeführten myokardialen „Mapping“-Techniken mit ihrer absoluten Quantifizierbarkeit magnetischer Gewebseigenschaften entscheidende Vorteile in der Charakterisierung früher vorklinischer bis terminaler Stadien myokardialer Erkrankungen [12].

#### T1-Mapping und extrazelluläre Volumenfraktion

**Figure 2:**
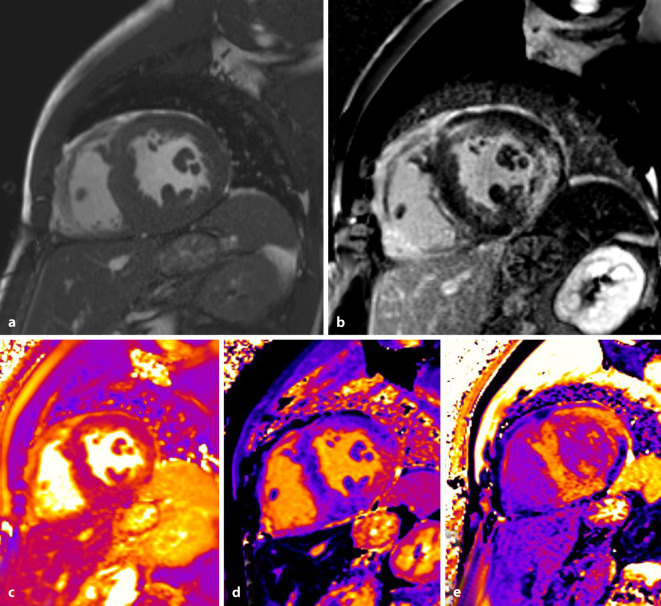

Myokardiales T1-Mapping bildet durch Akquisition multipler Inversions- oder Sättigungs-Erholungsbilder die T1-Relaxationszeit (longitudinale Relaxation) jedes Myokardpixels ab [12]. Zu den verbreitetsten und gebräuchlichsten Sequenzen zählen die modifizierte Look-Locker Inversion-Recovery Sequenz (MOLLI; [13]) und deren Weiterentwicklung („short“ MOLLI; [14]), welche die Dauer des Atemanhaltens verkürzen kann. In den typisch automatisiert errechneten T1-Maps des Myokards sind die pixelweisen myokardialen T1-Relaxationszeiten das Ergebnis des im Pixel befindlichen Anteils an Myozyten und Interstitium. Pathophysiologische Prozesse wie diffuse interstitielle Fibrose, Myokardödem oder Amyloidablagerungen zeichnen sich durch die Erhöhungen der T1-Relaxationszeiten aus, während Glycosphingolipid-Ablagerungen im Rahmen eines Morbus Fabry (Abb. 2) oder myokardiale Eisenüberladungen zu charakteristischen T1-Relaxationszeit-Verkürzungen führen [15]. Die Konsensuserklärung der Gesellschaft für kardiovaskuläre Magnetresonanztomographie (SCMR) empfiehlt auch für kommerziell erhältliche Pulssequenzen die primäre Verwendung lokaler Referenzwerte [12, 16].

Die Bewertung diffuser Erkrankungen soll durch eine Messung der ROI („region of interest“) im mittventrikulären Septum von Kurzachsenbildern erfolgen; bei fokalen Myokardprozessen sollten zusätzliche ROIs in Bereichen mit visuell abnormalem Erscheinungsbild erstellt werden [16].

Während die native T1-Relaxationszeit ein zusammengesetztes Myokardsignal aus Myozyten und Interstitium darstellt und den zugrundeliegenden Krankheitsprozess (Fibrose, Ödem, Amyloid, und/oder Myozytennekrose) alleine nicht vollständig differenzieren kann [17], repräsentiert die myokardiale extrazelluläre Volumenfraktion (ECV) einen weiteren mittels T1-Mapping bestimmbaren Parameter. Die Berechnung des ECV erfordert die Messung der T1-Relaxationszeit von Myokard und Blut vor und nach der Kontrastmittelapplikation. Zudem sollte der tagesaktuelle Hämatokritwert verfügbar sein, jedoch kann alternativ mit einer Hämatokritabschätzung anhand nativer T1-Werte des Blutes ein *synthetisches ECV* berechnet werden [16]. Um das für die Berechnung des ECV vorausgesetzte Gleichgewicht der Kontrastmittelkonzentration zwischen Blut und Interstitium zu erreichen, sollten mindestens 10 bis 30 min nach Kontrastmittelapplikation bis zur Akquisition der Kontrast-T1-Mappingdaten vergehen [10]. ECV-Normwerte gesunder Personen liegen im 1,5-T-MRT bei 25 ± 4 % und bei 27 ± 1 % in der 3‑T-MRT [18]. Anstiege des ECV werden hauptsächlich durch übermäßige Kollagenablagerung im Rahmen der Myokardfibrose verursacht oder durch Amyloidablagerungen, insbesondere im Subtyp der Transthyretin(ATTR)-Amyloidose [15].

#### T2-Mapping

Die Herausforderungen der bisher am häufigsten zur CMR-Ödemdetektion angewandten, konventionellen T2-gewichteten „short-tau inversion recovery“(T2-STIR)-Sequenz umfassen neben der Bewegungsartefaktanfälligkeit Artefakte, das – im Vergleich zu LGE-Sequenzen – intrinsisch geringe Kontrast-zu-Rausch-Verhältnis sowie den lediglich qualitativen bzw. semiquantitativen Ansatz dieser Technik [19]. Mittels T2-Mapping werden myokardiale T2-Relaxationszeiten als Surrogat des myokardialen Wassergehalts pixelweise quantifizierbar; darüber hinaus gilt T2-Mapping als reproduzierbarste aller CMR-Techniken der myokardialen Ödemerfassung [20]. Analog zum T1-Mapping wird die Akquirierung lokaler Referenzwerte empfohlen, die publizierten Normalwerte der T2-Relaxationszeiten unter Verwendung von „balanced steady-state free precession“(bSSFP)-Techniken liegen bei 52 ± 3 ms am 1,5-T-MRT und bei 45 ms am 3‑T-MRT [21, 22]. Eine bedeutende publikatorische Evidenz und teilweise entsprechende Leitlinien legen die Verwendung von T2-Mapping für folgende klinische Indikationen nahe: Myokarditis, Myokardinfarkt, Sarkoidose, Herztransplantatabstoßung und chemotoxische Kardiomyopathie.

### 4-D-Flussmessungen im Herz und den großen Gefäßen

Im Gegensatz zu 2‑D-Phasenkontrast-Flussmessungen, die etablierte Bestandteile klinischer CMR-Untersuchungen darstellen [23], erlauben 4‑D-Flussmessungen (d. h. zeitlich aufgelöste, EKG-getriggerte Aufnahmen nicht nur unidirektionaler, sondern aller 3 Geschwindigkeitskomponenten) eine wesentlich detailliertere Analyse der makroskopischen kardiovaskulären Strömungsmechanik.

**Figure 3:**
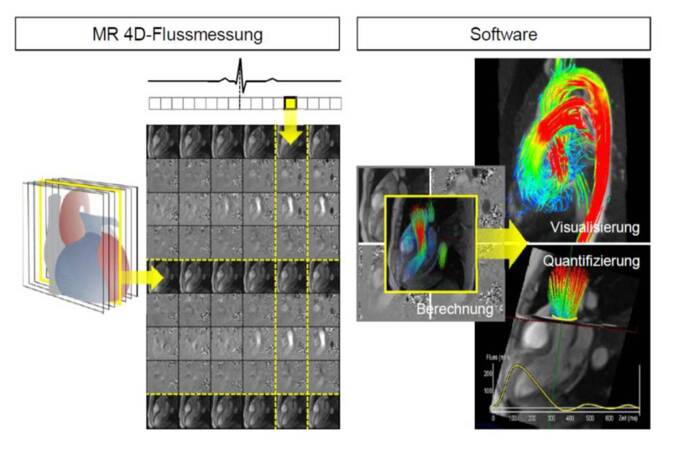

Die Aufnahmestrategie von 4‑D-Flussmessungen besteht typischerweise darin, das vollständige zeitlich veränderliche Geschwindigkeitsfeld innerhalb eines interessierenden Volumens unter freier Atmung zu akquirieren (Abb. 3). Dies kann entweder in Form von 2‑D-Mehrschicht-Cine-Phasenkontrastmessungen oder einer räumlichen 3‑D-Cine-Phasenkontrastmessung (welche die Gewinnung isotroperer Voxel erlaubt) geschehen [24]. Bei räumlichen Auflösungen der Größenordnung 2,5 × 2,5 × 2,5 mm^3^, der Abdeckung des Herzens und zeitlichen Auflösungen im Bereich von 40–60 ms liegt die Aufnahmezeit unter Ausnutzung von konventionellen Parallelakquisitionstechniken in der Größenordnung von 10 min; eine substanzielle weitere Beschleunigung scheint jedoch möglich [23]. Während für eine adäquate Analyse der Tausenden Bilder einer 4‑D-Flussmessung über lange Zeit ausschließlich Prototyp-Software zur Verfügung stand, sind mittlerweile unterschiedliche kommerzielle Lösungen erhältlich [25]. Damit werden Blutströmungen als Geschwindigkeitsvektoren, Stromlinien (Tangentialkurven an Geschwindigkeitsvektoren zu einer bestimmten Zeit) oder Teilchenpfade visualisiert und die Ermittlung von Flüssen durch beliebige Querschnittsflächen ermöglicht [24].

**Figure 4:**
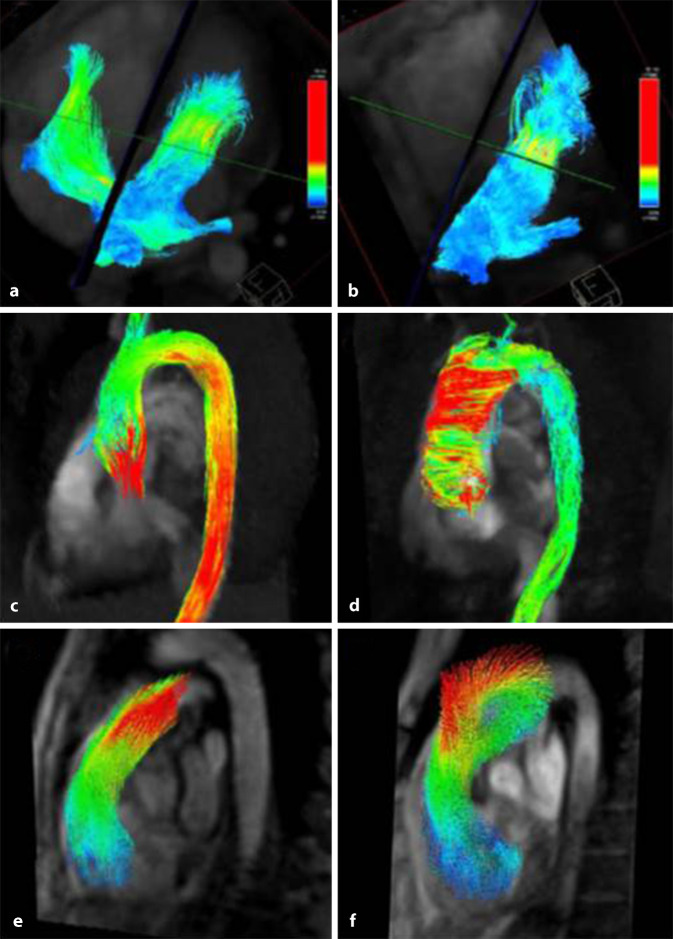

Potenzielle Anwendungen von 4‑D-Flussmessungen sind mannigfaltig. Verglichen zu multiplen 2‑D-Flussmessungen ist die Methode bei der Untersuchung angeborener Herzerkrankungen besonders attraktiv, da Messzeiten effektiv kürzer sind, Shuntvolumina genauer quantifiziert werden können und komplexe Strömungsverläufe zusätzlich visualisierbar sind ([26]; Abb. 4a). Auch die Genauigkeit der Bestimmung von Vorwärts- und Regurgitationsvolumina an Herzklappen lässt sich im Vergleich zu 2‑D-Flussmessungen verbessern, da sich die Querschnittsflächen, durch die hindurchtretenden Flüsse ermittelt werden, retrospektiv an die Klappenbewegung anpassen lassen [24].

Darüber hinaus könnten auch aus dem Geschwindigkeitsfeld abgeleitete, hämodynamische Größen diagnostische und prognostische Bedeutung erzielen:

1. Die turbulente kinetische Energie in der Aorta kann zur Echokardiographie ergänzende Information bei der Schweregradbeurteilung von Aortenklappenstenosen liefern [26]. Allgemeiner noch führen Veränderungen der Aortenklappe zu veränderten Strömungsverläufen (Abb. 4b) und regionalen Wandspannungen in der Aorta [26], wobei Letztere mit Veränderungen der extrazellulären Matrix der Aortenwand korreliert werden konnten [27].
2. Erhöhter pulmonalarterieller Druck führt zum Auftreten wirbelförmiger Strömung entlang des pulmonalen Hauptstamms (Abb. 4c), aus deren Dauer nichtinvasiv auf den pulmonalarteriellen Druck und dem Vorhandensein von Pulmonalhypertonie rückgeschlossen werden kann [28].
3. Metriken wie (turbulente) kinetische Energien, hämodynamische Kräfte, Konnektivitätskomponenten oder auch Wirbelbildungen in den Herzkammern zeigten Veränderungen unter systolischer und diastolischer Dysfunktion der Ventrikel oder bei Vorhofflattern [25, 26]; deren klinische Relevanz gilt es jedoch noch weiter zu untersuchen.

### Funktionsanalyse – innovatives Postprocessing

In der Analyse der biventrikulären Morphologie und Funktion gilt die CMR als Referenzstandard und die linksventrikuläre Ejektionsfraktion (LVEF) als der etablierteste Marker der globalen Myokardfunktion mit entscheidenden Auswirkungen auf das Patientenmanagement [29]. Frühere Tagging-basierte CMR-Messungen erwiesen sich als akkurat in der Erfassung des regionalen Myokardstrain, erforderten jedoch die Akquisition spezieller, zusätzlicher Sequenzen und eine aufwändige Nachbearbeitung, was deren routinemäßige Verwendung einschränkte [30].

Die neue Postprocessing-Methode des Feature Tracking (FT) ermöglicht nun die gleichzeitige Bewertung sowohl der linksventrikulären Ejektionsfraktion (LVEF) als auch des Strain unter Verwendung der standardisierten bSSFP-Cine-Bilder. Nach automatisierter endo- und epikardialer Grenzziehung werden – vereinfacht gesagt – aus der zwei- und dreidimensionalen Verschiebung kleinster anatomischer Elemente in sämtlichen Myokardsegmenten über den Herzzyklus die global longitudinale, zirkumferenzielle und radiale Deformation abgeschätzt [31]. Globale Strainwerte zeigten sich im Vergleich zu regionalen Strainwerten als robuster und reproduzierbarer [32]. Der global-longitudinale und der global-zirkumferenzielle Strain gelten dabei als konsistenteste Parameter, wobei generell global-longitudinale und -zirkumferenzielle Strainwerte von −20 bzw. −17 % als pathologisch angesehen werden [31]. In Patienten mit arrhythmogene rechtsventrikuläre Dysplasie (ARVD) konnte gezeigt werden, dass regionale rechtsventrikuläre Strainwerte die Detektion von Substraten ventrikulärer Tachykardie im Vergleich zur LGE-Technik verbessern [33]. In Patienten mit pulmonaler Hypertonie stellt der rechtsventrikuläre Strain eine vielversprechende nichtinvasive Alternative zur Beurteilung der Kopplung und diastolischen Funktion dar [34].

## Klinische Anwendungen

### Speichererkrankungen

In der Abklärung der Ätiologie eines hypertrophen LV ohne Vorliegen eines Aortenklappenvitiums oder einer Hypertonieanamnese kommt der kardialen MRT eine zunehmend bedeutende Rolle zu. Neben der Erfassung der Myokardmasse hat die differenzierte Analyse der Myokardstruktur eine hohe diagnostische und prognostische Relevanz. Hierbei können insbesondere die Speichererkrankungen Morbus Fabry oder eine kardiale Beteiligung im Rahmen einer Amyloidose unschwer diagnostiziert werden und zumeist gut von Formen der primären hypertrophen Kardiomyopathie unterschieden werden. Einen zentralen Stellenwert in der Diagnose nimmt hier das T1-Mapping vor und nach Kontrastmittelgabe ein [12].

#### Morbus Fabry

Der Morbus Fabry ist eine lysosomal vererbte Speichererkrankung mit einem α‑Galaktosidase-A-Enzymmangel, die am Herzen zu verschieden schwer ausgeprägten Einlagerungen von Glycophingolipiden in die Myozyten führt. Dabei entwickelt sich zunächst eine LV-Hypertrophie (LVH) und über begleitend inflammatorische Prozesse wird eine Narbenbildung induziert. Mit der Einführung quantitativer Mapping-Techniken wurde die bildgebende Frühdetektion einer kardialen Manifestation des Morbus Fabry möglich, da bereits in frühen Stadien der Erkrankung ein Abfall der T1-Relaxationszeiten unter 900 ms (1,5 T Feldstärke) beobachtet werden kann. Im späteren Verlauf der Erkrankung wird das myokardiale Ödem in den konventionellen STIR-Sequenzen sowie auf den quantitativen T2-Maps und eine zunehmende Narbenbildung im LGE charakteristisch (Abb. 2; [35]). Diese Prozesse werden primär bevorzugt im Bereich der basalen Lateralwand beobachtet und breiten sich danach auf den gesamten LV aus. Neben der Diagnostik einer kardialen Beteiligung bei M. Fabry eignet sich die multiparametrische CMR auch zur Verlaufskontrolle unter einer Enzymersatztherapie. Hierbei liegt der Fokus auf der Erfassung der Myokardmasse, dem T1-Mapping zur Graduierung der Lipideinlagerung sowie der Abklärung der myokardialen Inflammation mittels T2-Mapping [35].

#### Kardiale Amyloidose

Kardiale Beteiligungen im Rahmen einer systemischen Amyloidose sind insbesondere bei den Hauptformen der Leichtketten (AL Amyloidose) sowie dem Wildtyp (ATTR Amyloidose) zu beobachten. Hierbei kommt der Diagnostik einer kardialen Beteiligung eine essenzielle Rolle zu, da diese prognosebestimmend ist. Die Hauptziele der Bildgebung im Aufarbeiten einer suszipierten kardialen Amyloidose sind die Subtypisierung der Amyloidose sowie das Ausmaß des Befalls. Die weitere Typisierung erfolgt nuklearmedizinisch mittels Diphosphono‑1,2‑Propandicarbonsäure(DPD)-Knochenszintigraphie, welche eine Sensitivität von >99 % und eine Spezifität von 86 % für den Nachweis der ATTR-(Wildtyp‑)Amyloidose zeigt [36]. Patienten mit einer Leichtketten-Amyloidose zeigen typischerweise negative DPD-Knochenscans sowie eine monoklonale Gammopathie in den Laboruntersuchungen von Blut oder Urin [37].

**Figure 5:**
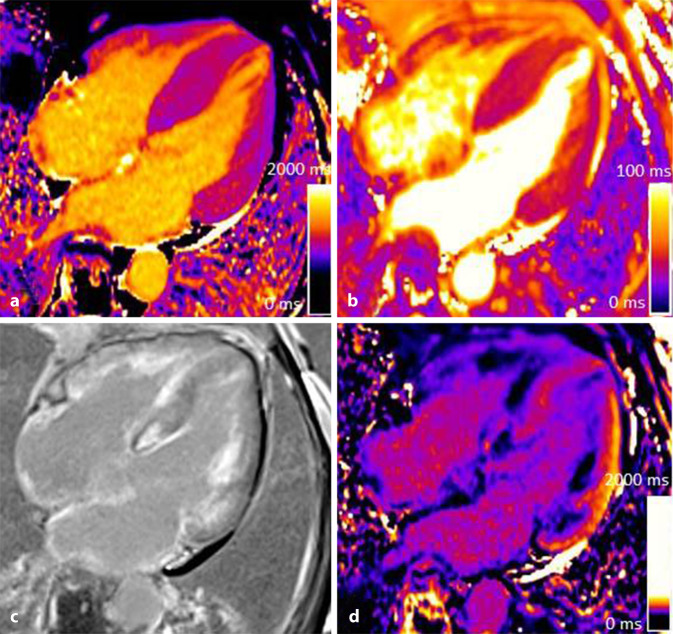

Die kardiale Beteiligung im Rahmen einer Amyloidose beruht auf einer pathologischen Amyloiddeposition im Extrazellularraum. In fortgeschrittenen Stadien zeigt sich ein restriktiver Phänotyp mit deutlicher LVH und biatrialer Dilatation sowie eingeschränkte myokardialen Strainwerte. Auch der rechte Ventrikel sowie die Atrien können diffuse murale Amyloideinlagerungen zeigen. Aufgrund dieser diffusen Amyloidspeicherungen gelingt die Myokardnullung typischerweise unzureichend, was die LGE-Bildqualität vor allem in späten Krankheitsstadien einschränkt. Mittlerweile hat sich T1-Mapping im Bereich der Amyloidoseabklärung sowohl diagnostisch als prognostisch etabliert (Abb. 5). Die T1-Relaxationswerte zeigen sich insbesondere in späten Krankheitsstadien deutlich erhöht (>1150 ms); dies schließt in Zusammenschau mit einem restriktiven CMR-Phänotyp andere Differenzialdiagnosen faktisch aus. Daneben hat sich eine Erhöhung des ECV als negativ prognostischer Parameter hinsichtlich der Überlebensrate etabliert [38]. T1-Mapping und ECV als Monitoring-Parameter in der Evaluierung neuer Therapiemöglichkeiten könnten in Zukunft zusätzliche Bedeutung gewinnen.

### Myokarditis/Inflammation

Die akute, infektiöse Myokarditis kann durch ein breites Erregerspektrum hervorgerufen werden und umfasst insbesondere Viren (Parvovirus B19, Adenoviren, Cocksackie etc.) und Bakterien (z. B. *Escherichia coli*). Infektiöse Myokarditiden können aber auch im Rahmen einer Tuberkulose, Autoimmunerkrankungen oder allergischer Geschehen auftreten oder medikamentös/toxisch induziert sein (z. B. durch Immunmodulatoren).

**Figure 6:**
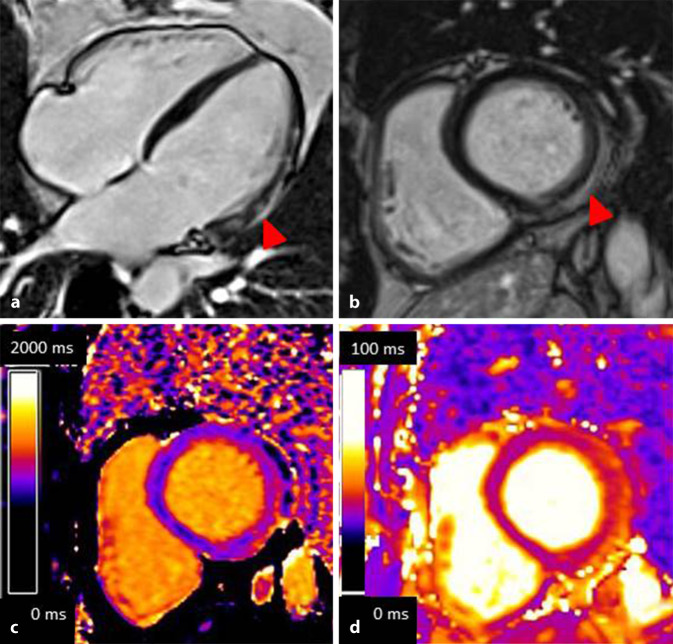

Die CMR-Diagnostik der akuten Myokarditis wurde seit 2009 durch die Lake-Louise-Kriterien definiert, welche 2018 durch die Berücksichtigung der Mapping-Technik aktualisiert wurden [39]. In der Myokardcharakterisierung mittels CMR müssen für die Diagnostik einer akuten Myokarditis jeweils ein T1- und ein T2-basiertes Kriterium erfüllt werden (Abb. 6). T1-basierte Kriterien entstammen aus dem nativen T1-Mapping oder dem typischen LGE sowie dem Post-Kontrast-T1-Mapping (ECV). Charakteristisch gelten dabei ein nichtischämisches Verteilungsmuster der Signalveränderungen (subendokardiale Aussparung oder ein nichtkoronares Bildmuster). T1-gewichtete Kriterien repräsentieren pathophysiologisch entweder eine Hyperämie und/oder eine Nekrosezone. Das T2-gewichtete Kriterium als Korrelat des Myokardödems und somit des akuten Geschehens entstammt dem quantitativen T2-Mapping oder der semiquantitativ erfassten Signalintensitäts-Ratio zwischen Myokard und Skelettmuskel ≥2 in konventionellen T2-gewichteten Bildern. Das Vorhandensein eines Perikardergusses, einer perikardialen Verdickung über 3 mm bzw. das Vorliegen einer reduzierten Ventrikelfunktion werden als supportive Befunde betrachtet, werden allerdings nicht zur Diagnose der akuten Myokarditis herangezogen.

Der Nachweis einer akuten Myokarditis in der kardialen MRT ist nicht unbedingt prognostisch wegweisend für den Patienten, da es nach Abklingen der Entzündung auch zur Restitutio ad integrum kommen kann [40]. Prognostisch bestimmend scheint vielmehr das Vorhandensein einer Narbe in einem Kontroll-CMR im Intervall zu sein [41].

Bei zahlreichen Systemerkrankungen aus dem rheumatischen Formenkreis, die mit einer generalisierten Inflammation einhergehen können, wurden in CMR-Studien subklinische Formen der kardialen Beteiligung nachgewiesen [42]. Hierbei lassen sich die für eine akute Myokarditis verwendeten Kriterien nicht immer zuverlässig anwenden, und die Zusammenschau mit klinischem Zustandsbild und Laborchemie (hs TropT, pro BNP) ist dabei wegweisend.

Im Rahmen der aktuellen COVID-19-Pandemie sind bereits kardiale Beteiligungen im Rahmen einer systemischen Inflammation oft prognostische hochrelevant. COVID-assoziierte Myokardiden wurden auch bereits mittels CMR sowohl im Akutstadium als auch nach durchgemachter Infektion nachgewiesen [43, 44]. Im Gegensatz zur *klassischen* viralen Myokarditis scheint nach Abheilung eine bevorzugte Narbenbildung im Bereich der Hinterwand vorzuliegen [43]. Erste Studien nach durchgemachter Erkrankung zeigen zudem, dass in 78 % der COVID-19-Patienten mittels CMR eine myokardiale Beteiligung nachgewiesen werden konnten, welche sich in mehr als der Hälfte der Fälle prolongiert darstellte [45].

### Ischämie

In den 2019 aktualisierten ESC-Guidelines zur chronischen koronaren Herzerkrankung (KHK) mit stabiler Angina pectoris wurden der nichtinvasiven bildgebenden Abklärung mittels Computertomographie (CT), CMR und nuklearmedizinischen Methoden eine wesentlich stärkere Rolle zugeteilt. Die koronare CT-Angiographie (CTA) zum Ausschluss einer KHK sollte demnach bei Patienten mit niedriger Vortestwahrscheinlichkeit durchgeführt werden. Für Patienten mit auffälliger CTA oder mittlerer Vortestwahrscheinlichkeit wird eine funktionelle Stresstestung mittels CMR oder äquivalenter nuklearmedizinischer Methoden zum Nachweis einer relevanten Myokardischämie vorgeschlagen; hierfür liegt in den aktuellen ESC-Leitlinienempfehlungen die Evidenzklassen IB vor [46].

Die mittels pharmakologischer Vasodilatation durch Adenosin oder Regadenoson bzw. unter Dobutamin-Belastung durchgeführte Stress-CMR-Untersuchung ist eine mittlerweile langjährig etablierte und standardisierte Methode zum Nachweis einer relevanten Myokardischämie. Die Wertigkeit der Stressperfusions-CMR wurde in den letzten Jahren durch mehrere große, prospektive Studien unterstrichen und ausgebaut. Die Studie *Clinical Evaluation of Magnetic Resonance Imaging in Coronary Heart Disease *(CE-MARC) zeigte die Überlegenheit der Stress-CMR im Vergleich zur Single-Photon-Emissions-Computertomographie (SPECT) in der Detektion einer signifikanten KHK [47]. Als Goldstandard wurde die invasive Koronarangiographie herangezogen, wobei die CMR mit 86 % vs. 66,5 % eine höhere Sensitivität und mit 90 % vs. 79 % zudem einen überlegenen negativen prädiktiven Wert im Vergleich zur Myokardszintigraphie zeigte. In CE MARC II zeigte sich eine Überlegenheit der Stress-CMR und der Szintigraphie gegenüber den National Institute for Health and Care Excellence (NICE) Guidelines in der Vermeidung nicht notwendiger invasiver KHK-Abklärungen [48]. Die erste vergleichende Wirksamkeitsstudie MR-INFORM zeigte zudem, dass ein auf nichtinvasiver CMR-Stressuntersuchung basierendes Patientenmanagement mit der invasiven intrakoronaren Druckmessung („fractional flow reserve“; FFR) bei Patienten mit stabiler Angina pectoris vergleichbar ist [49]. Die Stress-CMR zeigte sich im Beobachtungszeitraum von einem Jahr als genauso zuverlässig wie die invasive Untersuchung in dieser Hochrisikogruppe mit einer insgesamt niedrigeren Revaskularisierungsrate. Daneben konnte im Rahmen des sog. SPINS-Register gezeigt werden, dass Patienten mit negativer Stress-CMR-Untersuchung und fehlender postischämischer Narbe im 5‑Jahres-Verlauf eine sehr gute Prognose zeigen mit einer niedrigen Rate an kardiovaskulären Ereignissen von 0,6 %. Patienten mit zwei pathologischen Befunden zeigten ein Risiko von 3,5 % pro Jahr (*p* < 0,0001; [50]).

Zuletzt wurde die CMR in den 2020 ESC-Guidelines zum Management des akuten Koronarsyndroms auch eine zentrale Rolle im Management eines Myokardinfarktes bei nichtobstruktiven Koronararterien (MINOCA) zugeteilt [51]. Die Durchführung der CMR wird als entscheidende Bildgebungsmodalität in allen MINOCA-Patienten ohne offensichtlich zugrundeliegende Ursache mit einer Evidenzklasse IB empfohlen, mit dem Ziel, eine nichtischämische und ischämische Ätiologie zu differenzieren.

### Klappenerkrankungen

Die Echokardiographie stellt aufgrund ihrer breiten Verfügbarkeit, der Kosteneffizienz und der Möglichkeit, Blutfluss und Anatomie simultan zu erfassen, die Hauptmodalität in der Abklärung von Klappenerkrankungen dar. In Zusammenhang mit Klappenerkrankungen wird die CMR hauptsächlich zur Erfassung und Quantifizierung der Regurgitationsvolumina angewandt. Hier erfolgt die Messung über die Phasenkontrastangiographie in einer definierten Ebene nahe der jeweiligen Klappe. Insbesondere in der initialen Evaluierung und im Monitoring der Aorten- sowie Pulmonalinsuffizienz ist diese Methode aufgrund der guten Reproduzierbarkeit etabliert. Zudem können assoziierte Ventrikelveränderungen in Größe und Struktur genau erfasst und verfolgt werden.

Daten über die prognostische Wertigkeit der CMR-Erfassung der Regurgitationsfraktion sind derzeit noch limitiert [52] und auf Erwachsene mit angeborenen Herzfehlern (EMAH) im Bereich der Pulmonalklappe beschränkt [53]. Für EMAH empfehlen die 2020 ESC-Guidelines die CMR-Anwendung breit gestreut zur Erfassung von Anatomie, der Ventrikelfunktion, der Flussverhältnisse inklusive Shuntquantifizierung sowie die Stress-CMR für angeborene Pathologien der Koronararterien zur Erfassung einer relevanten Ischämie [54].

Neue Entwicklungen, insbesondere die 4‑D Flussmessung, werden in Zukunft weitere Einblicke in Klappenvitien assoziierte hämodynamische Veränderungen bieten. Die Erfassung von turbulentem Fluss (z. B. im Rahmen der pulmonalen Hypertonie oder bei bikuspiden Klappen) scheint hierbei von besonderer Relevanz zu sein [55].

## Fazit für die Praxis

- Das Indikationsspektrum der CMR (kardiale Magnetresonanztomographie) hat sich in den letzten Jahren kontinuierlich erweitert.
- Mit der Einführung quantitativer Mapping-Techniken sowie durch Resultate großer klinischer Ischämiestudien konnte die Wertigkeit der CMR in der Diagnostik und Prognostik deutlich erhöht werden.
- Damit fließen auch zunehmend mehr Empfehlungen zur CMR-Anwendung in die Guidelines der großen kardiologischen Gesellschaften ein.
- Die Kenntnis und Umsetzung dieser Leitlinienempfehlungen sind für die kardiovaskuläre Radiologie essenziell.
- Zukünftig werden technische Weiterentwicklungen wie beschleunigte Bilderfassung und auf künstliche Intelligenz gestützte Postprocessing-Analysen die CMR-Messzeiten und Bildauswertungen weiter beschleunigen und damit den Patientendurchlauf erhöhen sowie die Anwendung der Methode auch außerhalb von dezidierten Zentren erleichtern.
